# Clustering Time-Series Gene Expression Data Using Smoothing Spline Derivatives

**DOI:** 10.1155/2007/70561

**Published:** 2007-06-18

**Authors:** S Déjean, PGP Martin, A Baccini, P Besse

**Affiliations:** 1 Laboratoire de Statistique et Probabilités, UMR 5583, Université Paul Sabatier, Toulouse Cedex 9 31062, France; 2 Laboratoire de Pharmacologie et Toxicologie, UR 66, Institut National de la Recherche Agronomique (INRA), 180 Chemin de Tournefeuille, BP 3, Toulouse Cedex 9 31931, France

## Abstract

Microarray data acquired during time-course experiments allow the temporal variations in gene expression to be monitored. An original postprandial fasting experiment was conducted in the mouse and the expression of 200 genes was monitored with a dedicated macroarray at 11 time points between 0 and 72 hours of fasting. The aim of this study was to provide a relevant clustering of gene expression temporal profiles. This was achieved by focusing on the shapes of the curves rather than on the absolute level of expression. Actually, we combined spline smoothing and first derivative computation with hierarchical and partitioning clustering. A heuristic approach was proposed to tune the spline smoothing parameter using both statistical and biological considerations. Clusters are illustrated a posteriori through principal component analysis and heatmap visualization. Most results were found to be in agreement with the literature on the effects of fasting on the mouse liver and provide promising directions for future biological investigations.

## 

[1,2,3,4,5,6,7,8,9,10,11,12,13,14,15,16,17,18,19,20,21,22,23,24]
